# A geospatial method for estimating soil moisture variability in prehistoric agricultural landscapes

**DOI:** 10.1371/journal.pone.0220457

**Published:** 2019-08-21

**Authors:** Andrew Gillreath-Brown, Lisa Nagaoka, Steve Wolverton

**Affiliations:** 1 Department of Anthropology, Washington State University, Pullman, WA, United States of America; 2 Department of Geography and the Environment, University of North Texas, Denton, TX, United States of America; 3 Advanced Environmental Research Institute, University of North Texas, Denton, TX, United States of America; University at Buffalo - The State University of New York, UNITED STATES

## Abstract

Prehistoric peoples chose farming locations based on environmental conditions, such as soil moisture, which plays a crucial role in crop production. Ancestral Pueblo communities of the central Mesa Verde region became increasingly reliant on maize agriculture for their subsistence needs by AD 900. Prehistoric agriculturalists (e.g., Ancestral Pueblo farmers) were dependent on having sufficient soil moisture for successful plant growth. To better understand the quality of farmland in terms of soil moisture, this study develops a static geospatial soil moisture model, the Soil Moisture Proxy Model, which uses soil and topographic variables to estimate soil moisture potential across a watershed. The model is applied to the semi-arid region of the Goodman watershed in the central Mesa Verde region of southwestern Colorado. We evaluate the model by comparing the Goodman watershed output to two other watersheds and to soil moisture sensor values. The simple framework can be used in other regions of the world, where water is also an important limiting factor for farming. The general outcome of this research is an improved understanding of potential farmland and human-environmental relationships across the local landscape.

## Introduction

Studying the relationship between agricultural productivity and climate change has been important for evaluating culture change among many prehistoric societies (e.g., [1–5]). Agriculture is the engine that allowed for human populations to increase exponentially, and is associated with sedentism, urbanization, and increased social complexity. Higher crop productivity can support increasingly larger populations, which in turn need more food to be supported, also called the “agricultural treadmill” ([6], pp. 25–28). Alternatively, declines in agricultural productivity, particularly those brought on by climate change, have been linked to societal collapse and depopulation (e.g., [1–3,7–10]; and see also a summary of overshoot and collapse in [11]). In arid areas, drought can impact agricultural productivity, especially for dryland farming.

Changes in crop productivity have generally been studied using two approaches. The first is a large-scale, systems-based approach that incorporates a variety of variables including, but not limited to, climate, soil characteristics, infrastructure improvements, and nutrient supplements. These variables are analyzed together to understand shifts in crop productivity and its impacts on various populations, particularly under varying environmental conditions (e.g., [7,8,12–35]). These models are complex, requiring substantial statistical and programming skills and approaches. Such models have also been used to evaluate the applicability of modern data for assessing change in prehistoric contexts.

The second general approach for analyzing changes in ancient crop productivity focuses on the hydrological context and its impacts on crop yield (e.g., [29,36–41]), which is particularly important in arid areas where water is a limiting factor. Researchers have studied how water moves across the surface and how human-made features can help change the flow or infiltration of water. Because these types of hydrological models typically focus on surface water, they cannot be used to fully realize longer-term drought impacts on agricultural productivity. Subsurface hydrological processes are also important, particularly in dryland farming economies. Subsurface water can be stored for longer periods of time compared to water close to the surface. Hard or impervious surfaces do not allow for water to move and be stored in the subsurface soil. Water availability for plants is greatly reduced in volume and time when most of the water is surface runoff. For crops, when the focus is on surface water, underlying processes that provide longer term storage and availability of water are ignored.

To better understand how stored water might be retained in the soil during dry periods, we developed a model to estimate soil moisture variability across a watershed. Soil moisture relates to the amount of water present throughout a soil column rather than just at the surface ([42], p. 212; [43], pp. 1–2; and [44], p. 82). Given certain soil characteristics and topography, water is more likely to be retained in some areas than others. In this study, we use data on soil and topographic variables within a GIS analysis to characterize soil moisture potential across a watershed through the Soil Moisture Proxy Model (SMPM). A secondary analysis consists of applying the model to two additional watersheds, then comparing the output to real time soil moisture data. By comparing output to known soil moisture values, it is possible to validate the model, which we provide a preliminary example of in this paper.

**Fig 1:**
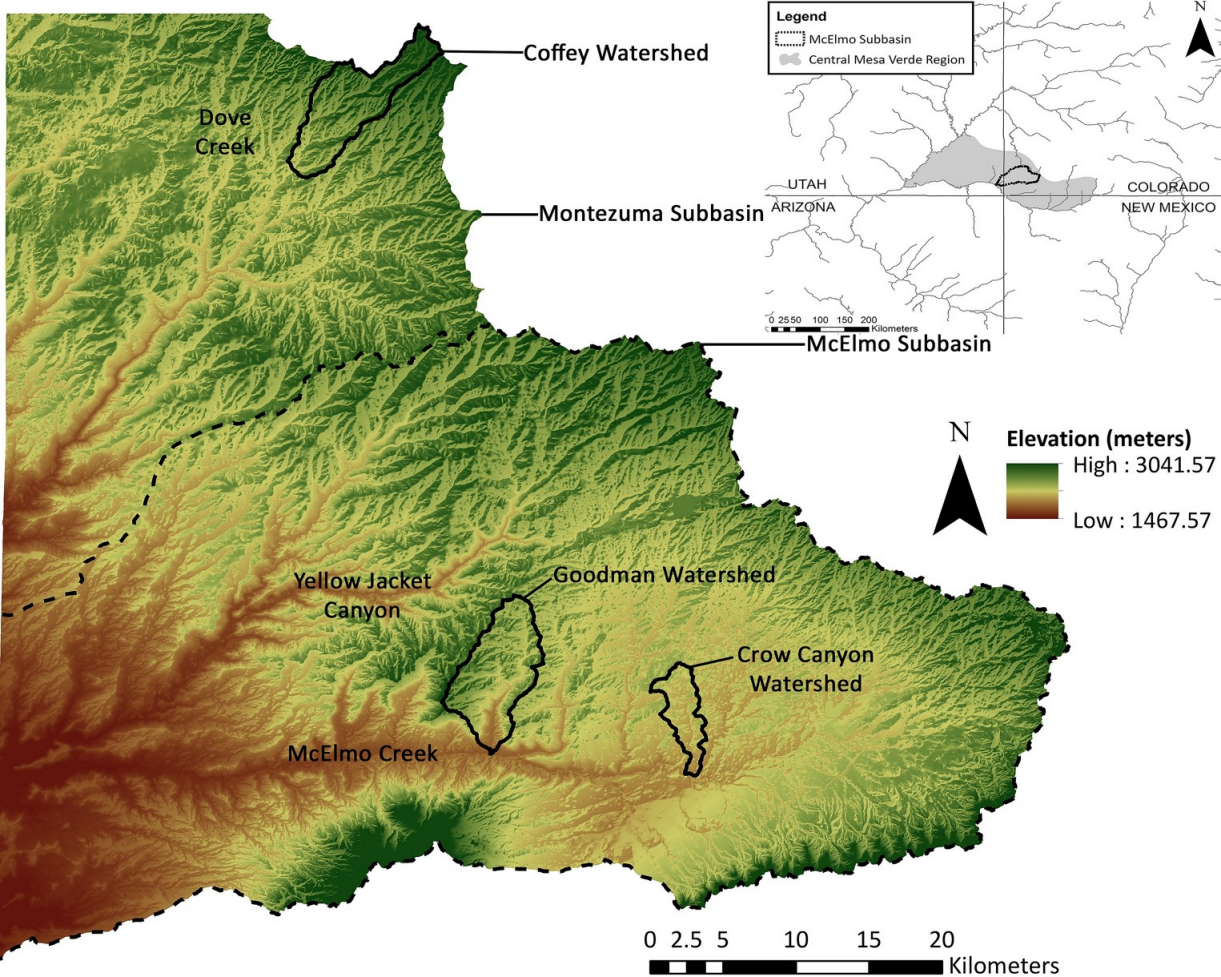
Digital elevation model (DEM) showing Goodman watershed and Crow Canyon watershed inside of the larger McElmo Subbasin (HUC-8), and the Coffey watershed inside of the Montezuma Subbasin (HUC-8). Inset (in the top right) showing the McElmo Dome Subbasin of southwestern Colorado and of the central Mesa Verde region. The data for the DEM was acquired from the USGS National Map Viewer (public domain).

We pilot this approach in watersheds in the central Mesa Verde region of southwestern Colorado (Fig 1). The rise and fall of the prehistoric population is well-known in this region (see summaries in [5,45]). For hundreds of years, the regional population increased and aggregated into pueblo communities, which were sustained by dryland agriculture. But by AD 1300, the region was depopulated, which is thought to be the result of a series of droughts that significantly decreased crop yield [4,7,46] playing out in a complex and geographically variable socio-political context [47]. Although the location and extent of prehistoric farmland could be estimated through surface features, such as terracing or water diversion features, etc., some scholars have examined changes in the soil or have used stable isotope analysis on maize cobs to identify farmland [29,30,48]. When these features are not present archaeologically or are difficult to see on the ground surface, then documenting changes in agriculture productivity is limited. The model presented here provides a different means for understanding the geographic distribution of farming soils related to soil moisture potential and thus crop yield, particularly under changing climatic conditions.

## Archaeohydrological research and modern hydrology

Researchers studying prehistoric agricultural systems have estimated productivity to understand its role in processes, such as population growth, sedentism, urbanization, violence, and social complexity (e.g., [5,12,17,19,21,25,26,35,49–51]). Agricultural productivity has been modeled using many different approaches. One method is to model an agricultural system or coupled human and natural system to demonstrate the linked causes and effects of culture and environment embedded in agriculture for a region [7,16,25,52]. For example, people may decide to build a checkdam, which causes sediment to build up, which enables people to farm and use that productive space. Scaling these and other seemingly small and potentially highly varied farming practices up to the regional scale, however, requires complex and large-scale models. As a result, such models, including climate-field reconstructions, de-emphasize farmer agency in particular locations (e.g., [53–56]), such as assessing the environment to choose locations of garden plots.

As an alternative, archaeohydrological models focus on a single limiting variable relevant to dryland farming, water. Soil water availability, however, is a complex, multivariate process, requiring modeling to assess the movement and retention of water, which helps to better understand its geographic variability and impacts on crop yield. In these models, water is simply a fluid that acts according to physical laws ([57], pp. 7–8; [58]; and [59], p. 1.1). The water balance equation used in hydrological studies incorporates several variables to model water inputs into and outputs from a column of soil [42,60,61]. The equation is as follows (given that density is constant):

$$\frac{dV}{dt}=(P+Q_{si}+Q_{gi})-(ET+Q_{so}+Q_{go}),$$ (Eq 1)

where $\frac{dV}{dt}$ is volume (V) of water storage for the time (t) rate of change, P is precipitation, *Q_si_* is the surface water inflow, *Q_gi_* is the groundwater inflow, ET is the evapotranspiration, *Q_so_* is the surface outflow, and *Q_go_* is the groundwater outflow [42,62]. Within one watershed, *Q_si_* and *Q_gi_* in Eq 1 are assumed to equal approximately zero, because defining boundaries for a catchment means that all water flows inside of that boundary and exit at one point. Therefore, for a catchment, Eq 1 can be simplified to the following:

$$\frac{dv}{dt}=P-ET-Q,$$ (Eq 2)

where precipitation (P) is the input into the water system, Q is surface and groundwater outflow, and ET represents the loss of water from the column.

Archaeohydrological models tend to focus on surface water flow or Q. Thus, such models may emphasize how water management systems (e.g., water control features) could have enhanced productivity, as well as how water availability affects agricultural systems (e.g., [29,36–39]). A limitation of these surface hydrological models is that they do not directly examine infiltration and retention of water within the soil column. An exception is Dominguez and Kolm’s [36] hydrological study, which used the water balance model to understand how soil moisture affects productivity in Hopi agricultural fields. They discussed variables related to both evapotranspiration and water movement (surface and subsurface) and how these can affect the amount of available moisture to plants. For example, a variable such as plant spacing could impact evapotranspiration and infiltration. When plants are closer together, plants will shade the surface more; thus, evaporation from the soil is lower. On the other hand, competition between plants for soil moisture increases with denser plantings. While this study demonstrated the importance of soil moisture for understanding crop yield, it did not provide a means to characterize soil moisture for a larger geographic area, which would enable consideration of the proximity of higher and lower quality cropland to particular localities, such as villages.

In modern agrohydrological studies, soil moisture is measured in two ways. First, it can be measured directly using sensors. This method is typically used for small areas such as a specific agricultural field to determine when to schedule irrigation [63]. To understand large areas and prehistoric agricultural systems, many sensors would be needed using this method to model soil moisture at the community scale, which would be expensive and logistically challenging. The second method is used at coarser scales and models soil moisture using proxy variables, such as soil properties and topography to assess evapotranspiration and runoff ([64,65]; [66], p. 5.2; and [67], p. 142). These two variables are incorporated into most agrohydrological models (e.g., [65,68–72]). Such models rely on the assumption that variables driving soil moisture are unlikely to vary significantly between modern times and the late prehistoric period and thus can be used to study archaeological contexts.

Three thresholds are used to demarcate the soil moisture available for plants to use: wilting point, field capacity, and saturation. Wilting point means that there is a low amount of soil moisture, causing a restriction in transpiration ([42], p. 243; [73–75]; and [76], p. 4.45). When soil moisture is at wilting point, the only water left in the soil is hygroscopic water, which adheres to the outer portion of a soil particle and is unavailable to plants. Thus, wilting point is the moisture threshold at which plants wither. On the other end of the spectrum is saturation, which occurs when all pore spaces are filled with water. Many crops require some air in the pore spaces to uptake nutrients and absorb water. Thus, saturated soils can also lead to plant or crop failure [77,78]. Crops such as maize can also become more susceptible to disease with prolonged saturation [79]. Field capacity is the range between wilting point and saturation and represents the amount of water that has remained in the soil two to three days after a rain event and after vertical drainage stops ([42], p. 213; and [76], p. 4.45). Field capacity is a balance between the vertical pressure gradient of water and the downward gravitational gradient. This is also generally the maximum amount of water that can be available to plants. Of these three, wilting point is likely to be the threshold of interest in arid environments.

In semi-arid and arid environments, water is the critical variable affecting success or failure of a crop. For archaeological settings then, a soil moisture model that uses two variables, soils and topography, would be particularly useful. The model would target soils and topographic variables that are the proxies for determining variability in soil moisture across the landscape—areas with higher and lower soil moisture. The model would not measure soil moisture directly, but would be predictive on an ordinal scale. It would provide a simplistic description of the landscape based on soil moisture.

## Modeling soil moisture

The SMPM describes the spatial variability of soil moisture across a watershed. We are not modeling water movement into, throughout, and out of the soil as in traditional hydrological models. Instead, we are holding precipitation constant and equal across the watershed. Without precipitation as an input, the model is a description of the potential for high/low soil moisture. Greater water retention is more likely to occur when ET and Q are lower (Eq 2). Thus, the locations where soil and topographic variables reduce ET and Q should have higher water retention and soil moisture than other areas. Two groups of variables, soils and topography, that impact ET and Q are used. The model uses one complex soil variable, plant available water (PAW), and two topographic variables, slope and solar radiation. PAW comprises a subset of variables that drive soil moisture, which is described below. Modern datasets for these variables are widely available at high resolution, except for the soils data, of which the limitations are further explained below. While vegetation can affect evapotranspiration, it is difficult to model prehistoric vegetation from modern data. Thus, in this model, vegetation is assumed to be constant and uniform across the watershed.

We apply our model within the Goodman watershed in the central Mesa Verde region of southwestern Colorado. The Goodman watershed covers approximately 33.25 sq km and is within a semi-arid climatic region—consisting of only a few substantial rainfalls per year [80,81] (Fig 1). In modern day, the annual rainfall is approximately 40 cm to 46 cm. Monthly precipitation is consistent throughout the year, averaging about 3.35 cm per month. However, the month of June decreases to approximately 1.25 cm [82]. Therefore, crops are heavily dependent on sufficient rainfall and snowmelt prior to June so that soil moisture is adequate for the agricultural growing season, which generally spans from approximately May 15 through September 2. Snowmelt is important in the region as it allows for a higher moisture content, thus helps provide moisture early in the growing season ([83], p. 19). The soils within the watershed consist mostly of loess, which is fertile and prime farmland for the region ([82,84,85]; [86], p. 213; and see also [87] for discussion on loess and [88]). Topographic variability in the watershed stems mostly from the change in elevation from the northwest to the southeast from the flatter area of the mesa into several small drainages and down into Goodman Canyon. The canyon widens and the slopes become steeper in the central and southern portions of the watershed. Elevation varies from approximately 1700–2185 masl. Most of the surface and groundwater that occurs in the watershed is constrained by topography, which inevitably drains into Goodman Canyon, and then into McElmo Creek at the southern point of the watershed.

Goodman watershed was chosen because of the extensive archaeological research conducted in the area (Fig 1). To delineate the Goodman watershed, we used the National Hydrography Dataset, which uses a hydrologic unit code (HUC) system that adds two digits every time the scale is reduced to a smaller sub-watershed, and spans from two (HUC-2) to 12 (HUC-12) digits [89]. The Goodman watershed is one of three sub-watersheds of the Trail Canyon-McElmo Creek watershed (HUC-12) within the McElmo Sub-basin (HUC-8) of the Upper Colorado River (HUC-2). The Goodman watershed boundary is not included in the HUC units as it is smaller than the HUC-12 digit. Thus, it was necessary to delineate the smaller Goodman watershed using a digital elevation model (DEM) [90] (see “Slope” section for DEM definition). Standard procedures were used to process and delineate the watershed by using the Hydrology toolbox in ArcGIS 10.2.2. ([91]; see also [92], p. 21). All underlying data are available and archived in Zenodo at https://doi.org/10.5281/zenodo.2572018.

### Plant available water (PAW)

Several individual soil variables (e.g., soil texture and depth to bedrock) could have been used in the SMPM. Instead, we chose the summary variable of plant available water (PAW), or Available Water Storage (AWS), which combines individual soil variables (i.e., soil texture and soil depth) and is a standard measure of the water holding capacity of the soil for plants. PAW is the maximum amount of water that can be stored within a given soil column from the ground surface to a maximum depth of 150 cm that is available to plants [93,94], and is a function of ET and Q. An area with a high PAW will have a greater amount of water at field capacity than an area with a low PAW.

PAW incorporates soil particle-size, which has a strong relationship to soil water characteristics [75,95]. Soil texture and particle-size affects ET and Q by influencing the pore space available for water to occupy, the rate at which water moves up, down, and across a soil column, infiltration, percolation [73–75], and capillary rise or the upward movement of moisture (i.e., increased upward movement of water to the surface can increase ET). Finer grained soils have high surface tension (i.e., lower saturated hydraulic conductivity or higher retention) and greater overall porosity, but smaller pore spaces between soil particles. So, while the soil can hold more water [96,97], water is also slower to infiltrate and percolate down through the soil profile. As a result, water can be available to plants for a longer period of time ([98], p. 17; and [99], p. 235). In contrast, in sandy soils, plants can rapidly uptake water by osmosis [100] immediately after a precipitation event, though the water drains away from the roots rapidly, thus, less water over time is available to plants. Therefore, a loamy soil that has closer to a third of clay, silt, and sand is more favorable to plants. Due to the strong relationship between particle-size and soil water, a standard available water capacity can be gained for each soil textural class following [66], Tbl 5.3.2 (Table 1) and see also [101–104].

**Table 1:** Available Water Capacity (AWC), and field capacity and wilting point volumetric percentages for different soil particle-size classes.

Soil Particle-size Classes	AWC at– 33 kPa, in cm^3^/cm^3^ ^A^^,^^B^	AWC at– 1500 kPa, in cm^3^/cm^3^ ^A^^,^^B^	Field Capacity (v%)^C^	Permanent Wilting Point (v%)^C^
Sands	0.02–0.16	0.01–0.06	10	5
Loamy sands	0.06–0.19	0.02–0.09	12	5
Sandy loams	0.13–0.29	0.03–0.16	18	8
Loams and very fine sandy loams	0.20–0.35	0.07–0.17	28	14
Silt loams	0.26–0.40	0.08–0.19	31	11
Silty clay loams	0.30–0.43	0.14–0.28	38	22
Sandy clay loams	0.19–0.32	0.09–0.21	27	17
Clay loams	0.25–0.39	0.12–0.28	36	22
Silty clays	0.33–0.44	0.19–0.31	41	27
Clays	0.33–0.47	0.21–0.34	42	30

^A^These columns in the table were reproduced from [66], Tbl 5.3.2. and see also [101].

^B^Hydrologists commonly use a threshold of -1500 kPa for calculating permanent wilting point [102,103] and -33 kPa for calculating field capacity ([104], p. 125; and [66], p. 5.11).

^C^The Field Capacity and Permanent wilting point columns are reproduced from [73].

PAW also incorporates soil depth, which is measured as depth to bedrock, but only to a maximum soil depth of 150 cm. Depth to bedrock of soils can affect the capacity and movement of water. Deeper soils can increase the volume and storage capacity of water in a soil column and provide more space for root growth, and thus may be more suitable for agriculture [36,105]. In addition, since maize root systems (the crop we are most interested in) are mostly above 150 cm, this depth is sufficient for assessing water storage across the landscape [106,107].

Soil particle-size and soil depth used individually do not account for water that percolates down through the soil profile due to gravity. PAW can be used to calculate the water capacity of a soil for plants with a specific texture and depth. As a summary variable PAW, thus, accounts for how rapidly water is likely to drain from a soil of given texture (or how much water the soil can hold after percolation slows down within a few days of a rain event), which is based in part on pore space. PAW summarizes the amount of water available between field capacity and wilting point (Table 1), and each soil type has a PAW number that reflects water volume. For example, sandy soils have low available water content relative to other soil types (Table 1). PAW is calculated by taking the soil water content at field capacity minus the permanent wilting point, which requires a series of calculations instead of a simple measurement. However, we downloaded PAW data directly from the Soil Survey Geographic Database (SSURGO) database (managed and curated by the United States Department of Agriculture Natural Resources Conservation (USDA NRCS)), which uses a weighted average from PAW (or AWS) values of the soil column (cm) [94] up to 150 cm in depth in the SSURGO data. AWS is calculated by multiplying the Average Water Capacity (AWC) for each soil class by the soil depth. SSURGO surveys consisted of measuring soils at one location, the results of which were then used to characterize soils in that class across large areas. Observation points from the soil survey were extrapolated and interpolated for the SSURGO maps. While not high resolution, this is the best dataset due to its finer resolution of 1:24,000, which was resampled to 10-m when converted to raster data. For reference of the 1:24,000 scale, one centimeter on the map is approximately 240 meters on the ground, or about 0.4167 mm on the map is approximately 10 meters on the ground. However, at a relative scale, soils with more sand are able to hold less water than soils with more clay; furthermore, deeper soils can retain more total water than shallow soils.

**Fig 2:**
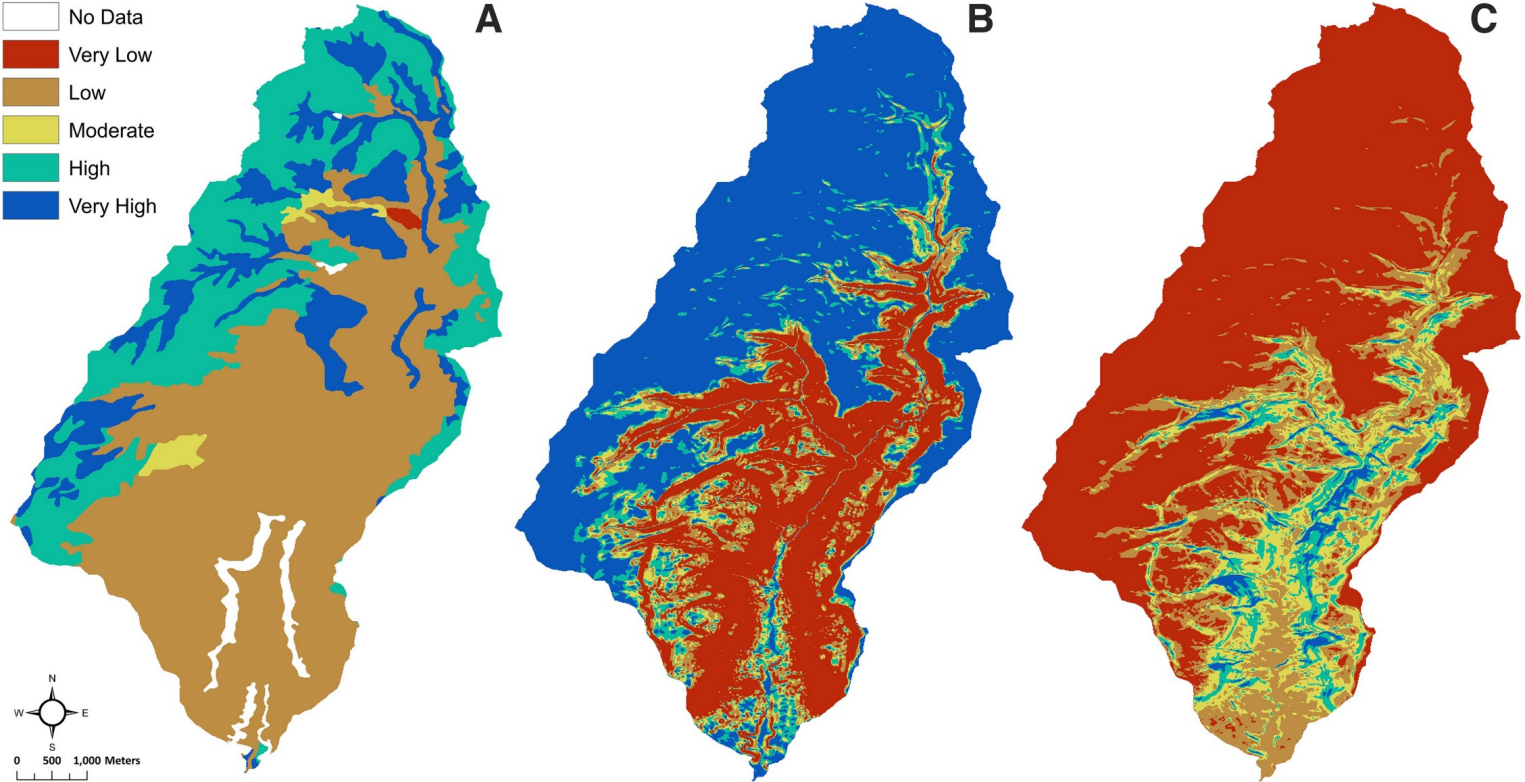
Classification of the three variables used in the SMPM for Goodman watershed. **(**A) The reclassification of plant available water (PAW) into five ranks, where very high represents the highest amounts of PAW, and the very low represents the lowest amounts of PAW. (B) The output shows the reclassification of slope into five ranks, where very high represents very low slope or a flat surface, and very low represents the steepest slopes. (C) The reclassification of the solar radiation (growing season) output into five ranks, where very high represents the lowest amounts of solar radiation, and the very low represents the highest amounts of solar radiation.

Here PAW has been reclassified into five ranks using the equal intervals method, so that there was an even distribution across the number of values (Table 2; Fig 2A; [108–111]; and [92], Appx. B.5). Generally, the NRCS uses an ordinal scale for PAW (e.g., very low to very high), and they divide PAW data using equal intervals. So, we also use equal intervals across the range of available values for any watershed for the description. A higher PAW value means higher potential soil moisture. The lowest cell values have shallow soils and a coarser soil texture. The highest cell values have deep soils and a finer soil texture ([92], p. 41). When PAW is greater, soil moisture is likely to be higher.

**Table 2:** Soil moisture proxy model summary for the Goodman watershed.

Model weights	Analysis criteria	Data category cut-offs	Input data and remarks
33.33%	Slope	10%	Percent slope was derived from the processed USGS 10-m resolution DEM [90]. Due to the lack of large structures, a DSM (digital surface model) was not used; however, this may be useful in the future as the study area expands.
		15%	
		20%	
		30%	
		> 30%	
33.33%	Plant available water (PAW) (in cm)	≥ 22.348	PAW (or Available Water Storage) was derived from the SSURGO soils data (downloaded on September 10, 2018^A^). PAW was extracted using the ArcGIS Soils Data Viewer add-in [94,110,111]^B^. Data category range is 27.680 to 1.020.
		17.016	
		11.684	
		6.352	
		1.020	
33.33%	Solar radiation (WH/m^2^)	≤ 596187.133	Solar radiation was derived from the USGS 10-m resolution DEM (United States) [90], and was calculated using the ArcGIS tool (solar radiation) in the Spatial Analyst toolbox. Since the focus is on agriculture, the radiation was averaged for the entire growing season approximately Day 135 to 245. Data category range is 596187.133 to 801829.688.
		667794.808	
		721041.541	
		763271.709	
		801829.688	
100%	Soil moisture proxy model	≥ 10 (Very high soil moisture)	Sum of the three variables listed above, and reclassified using natural breaks. Three is the lowest value represented. Bedrock was excluded as no data. Data category range is 12 to 0.
		8 (High soil moisture)	
		6 (Moderate soil moisture)	
		5 (Low soil moisture)	
		0 (Very low soil moisture)	

^A^https://websoilsurvey.sc.egov.usda.gov/DSD/Download/Cache/SSA/wss_SSA_CO671_soildb_US_2003_[2018-09-10].zip.

^B^We used the Soil Data Viewer extension from the USDA NRCS in ArcGIS instead of using the ESRI SSURGO Downloader, which had some value differences from the USDA NRCS. Therefore, the USDA NRCS data is used as the authoritative source for soils data.

### Slope

PAW summarizes soil characteristics that drive soil moisture, but topography also plays a role and must be incorporated into the SMPM. Slope refers to the steepness of the change in elevation, which affects runoff (Q). For example, the steeper the slope, the more likely that precipitation will move downslope rather than infiltrate into and percolate through the soil [112,113]. To measure slope, we use DEMs. As raster datasets, DEMs are samples of elevation at different intervals (e.g., 10 m) across the landscape ([114]; and [115], pp. 7–8). Ten-meter resolution DEMs were downloaded from the National Elevation Dataset of the United States Geological Survey (NED USGS) [90], which were processed ([92], Appx. A.1 for process) to cover the McElmo Sub-basin, Goodman watershed, and Montezuma Sub-basin (Fig 1). The DEM and other variables below were processed using ESRI’s ArcGIS 10.2.2 software. The NED’s 10-m resolution data are the finest resolution for the McElmo Sub-basin, since no LiDAR data are currently available [90]. From the DEMs, we calculated the percent slope surface, the amount of elevation increase (or rise) divided by the distance of the slope (or run) multiplied by 100 [116–118]. During the process of calculating slope, it was necessary to determine the z-factor, which was done by relying on the latitude of the watershed. The z-factor adjusts for differences of vertical measurement from the horizontal measurement (x, y) across space. The latitude is approximately 37 North for the watershed; so, we rounded to 40 to use the 0.00001171 value provided by [119].

The percent slope data are reclassified into five ordinal groups (Table 2, Fig 2B). Slope groupings are ranked one to five, with five representing the lowest slope and the greatest likelihood for infiltration and higher soil moisture. The cutoff points for the rankings were chosen based on previous successful analyses [23,24,49,120], the overall distribution of slope values for the Goodman watershed, and based on the physics of water and slopes—the severity of surface runoff increases with the increase in the slope percentage [121]. Essentially, 10% was at one standard deviation for the slope data; therefore, we used 0–10% as the range for the lowest runoff potential. Lateral water movement is low below 10%. We chose the 10–15% range because this was the next step for a one-half standard deviation. Thirty percent was the value for two standard deviations for the slope data. We split the 15–30% range into two categories (15–20% and 20–30%). Slope varies a lot in the 10–20% range; therefore, we used a 5% increment for the middle ranks (i.e., two and three). Greater than 30% was used for the category with the greatest runoff potential.

Slope variability in the Goodman watershed is dominated by the canyon and mesa features. Most of the slopes greater than 15% are associated with canyon walls, while the areas with lower slopes are located on the mesa and on the canyon floor. The areas with very high and high potential to retain soil moisture tend to be located on the mesa, with the slope variability associated with the smaller hills and drainages.

### Solar radiation

Slope and its relationship to runoff potential represents one impact of topography on soil moisture potential; a second topographic variable that must be considered is solar radiation, which affects ET and is derived from aspect. Aspect is the direction a slope is facing that is typically used as a measure of sun exposure [116,122,123]. Generally, for the northern hemisphere, incident solar shortwave radiation is higher on a southern aspect compared to northern aspects (e.g., [124–129]). As a result, north facing slopes experience lower evapotranspiration (e.g., [130], pp. 70–71; and [127,131,132]). However, since aspect generally assumes an overhead sun, it does not account for the variability in sun angle for different latitudes and seasons. Thus, solar radiation is an alternative measure that incorporates sun angle and solar intensity for different latitudes and times of the year ([42], p. 41); [133], pp. 18–66); [134], p. 255; and [135]). For example, during the summer (and the growing season), the sun is higher in the sky and exhibits more of a direct angle to the surface and thus greater solar radiation, producing higher evapotranspiration. Solar radiation is in the SMPM to determine what areas receive the highest amounts of radiation and have the greatest potential for evapotranspiration.

We averaged solar radiation across the growing season (start day 135 to end day 245) for the Goodman watershed using the ArcGIS Area Solar Radiation tool [136]. We used the 10-m resolution DEM [90] as the input raster to calculate solar radiation. We reclassified the data into five ranks using the natural breaks method (Table 2; Fig 2C; [92], Appx. B.4). The natural breaks (Jenks) classification is defined by ESRI [137] as “a method of manual data classification that seeks to partition data into classes based on natural groups in the data distribution. Natural breaks occur in the histogram at the low points of valleys. Breaks are assigned in the order of the size of the valleys, with the largest valley being assigned the first natural break” [138,139]. The natural breaks method allows capture of variability through intensional rather than extensional (imposed) classification [140]. For this analysis, classifying by the natural breaks method created classes with an even representation of solar radiation. The very high category represents higher potential for soil moisture retention because of low solar variability, and thus lower ET. The very low category represents lower potential for retaining soil moisture because of greater solar radiation and ET.

Within the Goodman watershed, solar radiation variability is largely due to the small hills, drainages, and the canyon (Fig 2C). Shaded canyon escarpments have the lowest solar radiation and thus, high to very high soil moisture potential, while mesa areas have the highest solar radiation and low to very low soil moisture potential.

## Soil moisture proxy model (SMPM)

**Fig 3:**
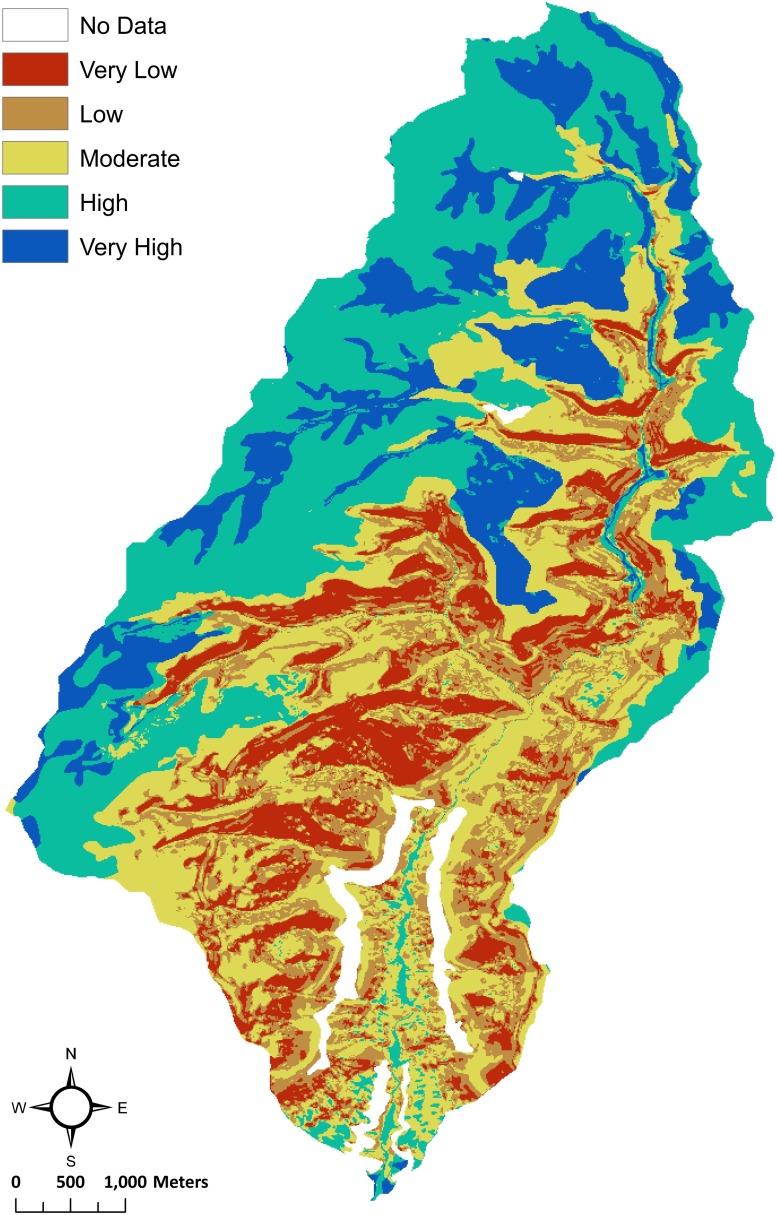
Soil moisture proxy model results for Goodman watershed. The output (as well as the output for the additional watersheds) shows that the very high rank represents very high soil moisture, and very low represents the lowest soil moisture areas.

The SMPM takes each of the three variables (PAW, slope, and solar radiation) described above and compiles them into a geoprocessing model representing the potential soil moisture distribution at an ordinal scale (Table 2). The variable ranks were combined using ArcGIS raster calculator to sum the ranks: Slope + Solar Radiation + PAW [23]. Cells with zero are exposed bedrock/rock outcrops or water and were removed from analysis. The sums ranged up to 12, which were then classified into five ranks using natural breaks across the output (Fig 3). The purpose of the categories is to reflect low to high soil moisture potential across the watershed.

For the model output, the higher cell values indicate lower ET and Q, thus, soil moisture is expected to be higher (Eq 2). A very high ranking means a low slope percentage, low solar radiation, and high PAW (usually deeper soil depth and silty-loam soil), meaning that water will be more likely to leave the root zone slower (i.e., soil moisture loss is lower) than for a pixel with a very low ranking. A very low ranking pixel would have a high slope percentage, high solar radiation, and lower PAW (usually a sandier soil), meaning that water will be more likely to leave the root zone quicker (i.e., soil moisture loss is greater) than for a pixel with a very high ranking.

Since the three variables (i.e., slope, solar radiation, and PAW) are equally weighted in the model, here they have an equal influence in driving variability in soil moisture potential across the watershed. Forty-five percent of the watershed has very high and high soil moisture potential (Table 3). These soils are located on the mesa and in the canyon bottom, which tend to have very high PAW and very low slope. In contrast, the canyon slopes have moderate to very low soil moisture potential because they are steep, over 15% grade, with very low PAW due to shallow soil depth.

**Table 3:** Soil moisture proxy model summary of the area (m^2^) of soil moisture and percentage of Goodman watershed (GW), Goodman watershed (GW) mesa-only, Crow Canyon watershed (CCW), and Coffey watershed (CW) of the five ranks. Area and percentages exclude the “no data” portions of the modeled watersheds.

	Goodman Watershed		GW–Mesa-only		Crow Canyon Watershed		Coffey Watershed	
Availability Rank	(Area) m^2^	% of GW	(Area) m^2^	% of GW—mesa	(Area) m^2^	% of CCW	(Area) m^2^	% of CW
Very low (1)	4,056,158	12.5	554,994.99	3.1	808,728	7.3	2,588,220	8.5
Low (2)	5,379,381	16.6	1,388,380.03	7.8	881,945	8.0	3,588,649	11.7
Moderate (3)	8,298,059	25.6	3,340,948.43	18.6	2,539,609	23.0	5,545,696	18.1
High (4)	10,015,616	30.9	8,336,081.83	46.5	4,545,010	41.2	5,947,960	19.4
Very high (5)	4,655,871	14.4	4,293,758.26	24.0	2,268,776	20.5	12,932,699	42.3

Several decisions can affect the mapped output of the model that researchers need to consider when implementing the model. These include the number of ranking intervals, the method used to create breaks in data, and the variability in the landscape. First, we experimented with using different numbers of classes (3 to 10). While a higher number of classes created more variability primarily on the mesa in the Goodman watershed and divided the very high soil moisture patches into smaller ones, the small differences between, for example, an eight and ten ranking did not appear to be meaningful in our analysis. For example, a PAW value of 27 (10 ranking) and a 24 (8 ranking) would still be very high (Table 2). Thus, we used fewer classes allowing us to capture larger patches of very high soil moisture, which are likely to be the best farmable land. Second, we sought to separate some of the variable ranks in effective ways. We chose from a variety of different methods (e.g., equal intervals, quantile, natural breaks, and standard deviation) that are available in ArcGIS. Third, the variability of soils and topography across the landscape and the heterogeneity of the landscape also affects the decision for the number of classes. The rankings for the variables could vary by context and region. For example, when there is a wider array of values that are not outliers, then more classes could be used for the variables. Smaller interval sizes for the ranked classes would likely still produce a map output with larger patches, unlike for Goodman watershed. On the other hand, if an area is relatively homogeneous, then a researcher might want to use fewer categories. A larger range of values for each variable would produce larger patches. If more classes were used for a homogeneous landscape, then the output is unlikely to change much. Additionally, very minor differences between values could produce a higher ranking for a pixel, when in reality that pixel is very similar to other ones of lower rank. Ultimately, there is flexibility in how variability can be handled in the model, including subsets of a watershed and numbers of classes. In summary, when classifications are adjusted, the resolution of output is different, and the three variables react differently to reclassification.

**Fig 4:**
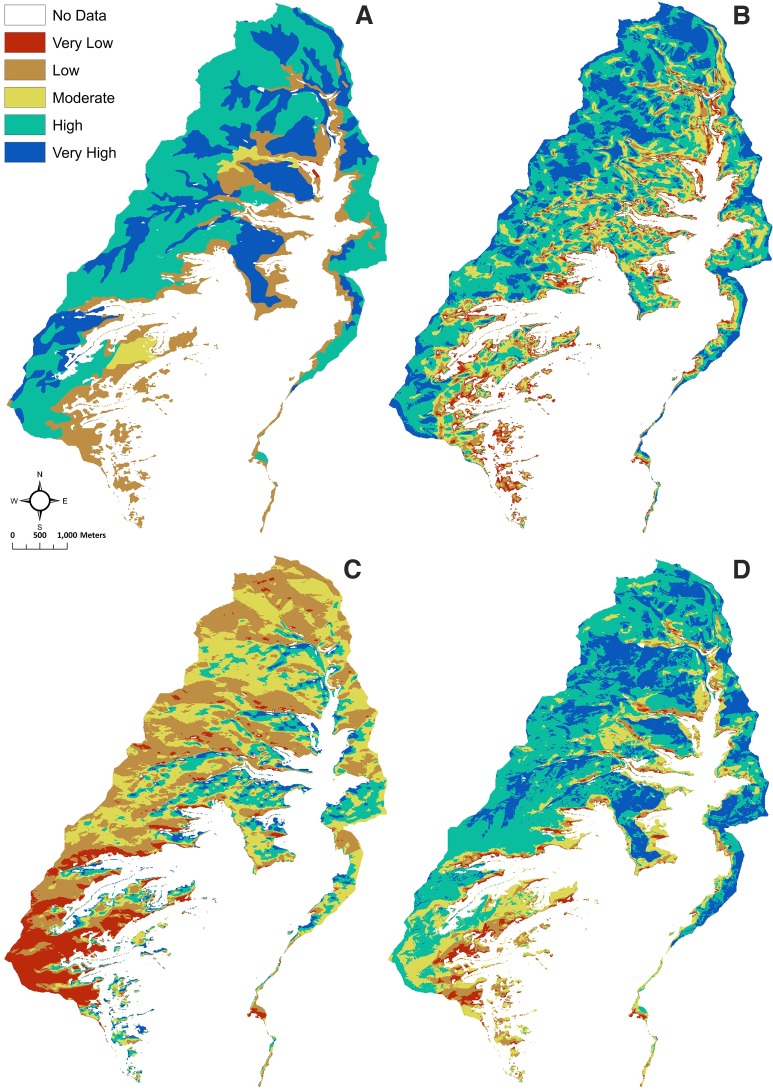
Model results for Goodman watershed mesa-only. (A) The reclassification of plant available water (PAW) into five ranks, where very high represents the highest amounts of PAW, and the very low represents the lowest amounts of PAW. (B) The output shows the reclassification of slope into five ranks, where very high represents very low slope or a flat surface, and very low represents the steepest slopes. (C) The reclassification of the solar radiation (growing season) output into five ranks, where very high represents the lowest amounts of solar radiation, and the very low represents the highest amounts of solar radiation. (D) The output shows that the very high rank represents very high soil moisture, and very low represents the lowest soil moisture areas.

To illustrate how the decision-making process can affect the output of the model, we analyzed a subset of the watershed. Since the canyon slopes, in particular, are not likely to be farmed, we reanalyzed the data by excluding the area represented by the canyon and focusing only on the mesa top (Fig 4). The boundary of the subset area was set by using thresholds of less than 15% slope and greater than 2015 m for elevation, which is the lowest elevation at the edge of the mesa. This effectively excludes the area with low PAW in the original output (Fig 2A). Since the slope classification had to be changed from the original model, Equal Interval reclassification is an appropriate choice for capturing some variability on this smaller scale (Fig 4B).

In the mesa-only model, reclassifying the PAW only affected a few locations of very high soil moisture, indicating there is little variability in PAW on the mesa. In contrast, the reclassification of slope and solar radiation resulted in more variability across the mesa (Fig 4B and 4C). The effect of documenting this smaller scale variability is that the higher moisture areas increased in proportion while the areas classified as lower soil moisture decreased (Table 3). In the whole watershed model, the variables equally drive the location of larger patches of very high soil moisture on the mesa, although slope and solar radiation contribute some variability on the mesa and even more so in the canyon. Since the mesa-only model is a higher resolution model, it picks up more variability of slope and solar radiation, which contribute more to the patchiness and divergence between high and very high soil moisture. Alternative strategies could have been used, such as increasing the number of categories. However, the mesa and canyon provided a discrete spatial boundary that corresponded to higher slope, lower PAW, and lower solar radiation, which provides a micro-geographic basis for our approach. If in other cases there is not a clear way to create a spatial subsample, then increasing the categories is a way to increase resolution.

## Evaluation of the SMPM

To evaluate the accuracy of the model, we compared soil moisture data to the model output. Two methods can be used for validation of the model. First, land surface temperature data from MODIS (MODIS Land Surface Temperature and Emissivity) [141] are sometimes used in conjunction with vegetation indices [142–144] as estimates of soil moisture. Ground and surface temperature have a strong positive correlation, whereas, soil moisture and surface temperature have a negative correlation [145], which may also vary seasonally [146,147]. The resolution of this method is approximately 1-km rather than the 10-m resolution used in our model. Thus, we would only be able to evaluate the accuracy of the model for very large areas. Given that we sought to develop a method for documenting soil moisture variability at the scale that a prehistoric farmer would be interested in, this validation method would not be useful. Additionally, researchers use several different methods to derive soil moisture from surface temperature data, which can lead to different results [148–150]. Another option for model validation is to use soil moisture data collected from sensors. As point data, the sensors have the advantage of being at a scale closer to that of our model than to that of the remote sensed data. The disadvantage is that such data can only be used to validate the model at the scale of individual pixels.

Given that the resolution of the surface temperature data was too coarse, we used soil moisture sensor data to provide a pilot evaluation of the model output. Each sensor comprises a data logger (Decagon EM50 Digital Data Logger) and three plug-in sensors (5TM Soil Moisture and Temperature Sensor) that were placed at depths of 15 cm, 30 cm, and 45 cm below the ground surface. The 5TM sensors take hourly measurements of volumetric water content (m^3^/m^3^) and temperature using a thermistor. The moisture values were assessed over the course of three growing seasons from May 23 to September 2, 2015 and May 15 to September 2, 2016 and 2017.

**Fig 5:**
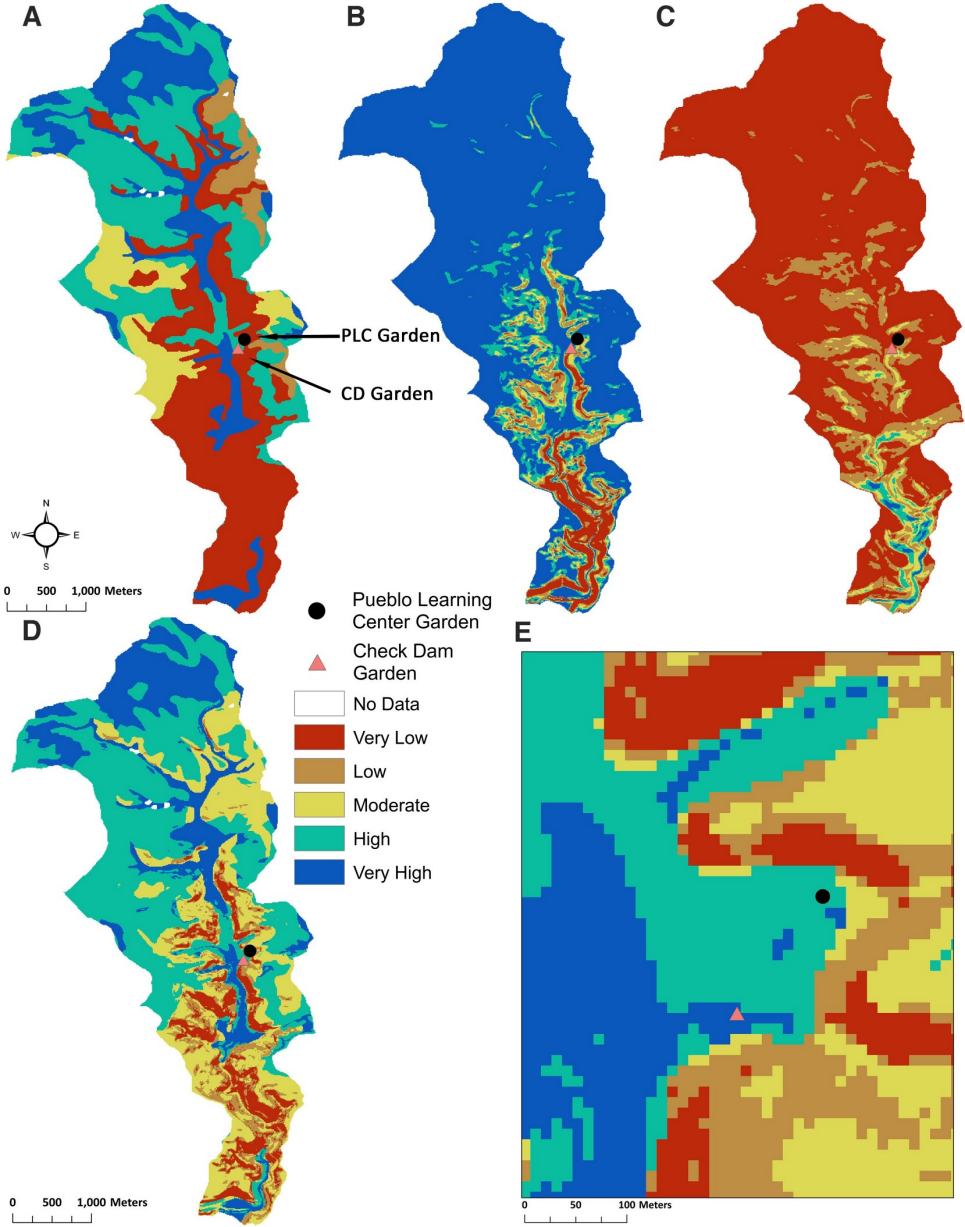
Classification of the three variables used in the SMPM and the SMPM results for the Crow Canyon watershed with close ups of the Pueblo Learning Center (PLC) and Check Dam (CD) gardens. (A) Reclassification of plant available water (PAW). The arrows show the location of the soil moisture sensors in the Pueblo Learning Center (PLC) and Check Dam (CD) gardens. (B) Reclassification of slope. (C) Reclassification of solar radiation (growing season). (D) Soil moisture proxy model results. (E) Zoomed in version of D, showing that the Check Dam garden is in the very high soil moisture, and the Pueblo Learning Center garden is in the high soil moisture.

Since we were unable to place sensors in the Goodman watershed, we placed sensors in experimental maize gardens in a nearby watershed (the Crow Canyon watershed) and an agricultural field under dryland bean cultivation in the Coffey watershed. The Crow Canyon and Coffey watersheds were modeled individually using the SMPM to determine whether the higher soil moisture sensor data corresponded with the SMPM. The model was constructed and run using the exact same procedures and parameters as was modeled for the Goodman watershed. The Crow Canyon and Coffey watersheds do not have the steep canyon topography like Goodman watershed. These two watersheds are more of a typical riparian landscape, which makes slope a less dominant variable. The three watersheds (i.e., Goodman, Crow Canyon, and Coffey) are approximately at the same scale (i.e., below HUC-12). Two experimental gardens are in the Crow Canyon watershed, which is located along McElmo Creek and is in the same subbasin as the Goodman watershed (Fig 1). The Crow Canyon watershed sensor values likely represent a similar soil moisture context as that in the Goodman watershed, given relative proximity of the two areas (Fig 1). Sensors were placed in the Check Dam and Pueblo Learning Center gardens (Fig 5) set up by Crow Canyon Archaeological Center as part of the Pueblo Farming Project [17] (see also https://crowcanyon.github.io/pfp_ebook/). The Check Dam garden is located slightly upslope from the valley bottom at an elevation of about 1867 m and gets its name from the prehistoric feature within the plot that appears to have been used to direct water flow. Three sensors were placed in this garden and average values were used. One sensor was placed in the Pueblo Learning Center garden located upslope near the mesa top at an elevation of about 1872 m. For the Crow Canyon watershed, the spatial distribution of very high to very low potential soil moisture is similar to the Goodman watershed. However, Crow Canyon watershed has a greater proportion of high soil moisture area and the Goodman watershed has a greater proportion of very high soil moisture area.

The SMPM output shows that the Check Dam garden is within a very high potential soil moisture area, whereas, the Pueblo Learning Center garden is in a high potential soil moisture area (Fig 5D and 5E). Thus, it is expected that the Check Dam garden will have higher soil moisture than the Pueblo Learning Center garden across all depths. Some differences such as vegetation density can also be seen on satellite imagery. The collected soil moisture data reveal that the Check Dam garden has higher soil moisture at all depths (Table 4). Although this evaluation can only be considered preliminary due to small spatial coverage and limited sampling, the soil moisture data from the Crow Canyon experimental plots reveal that in this case our model documents relative differences in soil moisture (Table 5).

**Table 4:** Collected soil moisture data (m^3^/m^3^ –volumetric water content (VMC)) and temperature data (celsius) from five soil moisture sensors in the Crow Canyon and Coffey watersheds. The values from the three sensors in the Check Dam garden were averaged together. The values represent averages of hourly data from May 23, 2015 to September 2, 2015 and May 15 to September 2, 2016 and 2017, which was treated as the agricultural growing season. The soil moisture sensors were placed at 15 cm, 30 cm, and 45 cm below the ground surface.

Garden	Watershed	n	Volumetric Water Content (m^3^/m^3^)			Temperature (Celsius)				
			15 cm	30 cm	45 cm	15 cm	30 cm	45 cm	VMC Avg	Temp Avg
Pueblo Learning Center	Crow Canyon	7785*	0.138	0.144	0.156	23.1	22.1	21.2	0.146	22.1
Check Dam	Crow Canyon	22020	0.197	0.213	0.184	23.0	22.2	21.3	0.198	22.2
Coffey	Coffey	7542	0.204	0.228	0.295	20.2	19.1	18.6	0.243	19.3

*Exception to sample size: For the 15-cm level, the cable that connects to the data logger came partially unplugged and did not record data from March 16, 2017 (2:00 pm) through June 7, 2017 (8:00 am); therefore, the sample size was reduced to 7224.

**Table 5:** The SMPM and volumetric water content (m^3^/m^3^) for the three garden locations, with the three variables, plant available water (PAW) (in cm), solar radiation (WH/m^2^), and slope, in the model.

Garden	Watershed	PAW	Slope	Solar Radiation	SMPM Output	Soil Moisture Characterization	Average Volumetric Water Content
Pueblo Learning Center	Crow Canyon	21.51	9.25	771561.94	10	High	0.146
Check Dam	Crow Canyon	21.51	5.44	758261.19	11	Very High	0.198
Coffey	Coffey	26.90	8.39	789948.06	12	Very High	0.243

**Fig 6:**
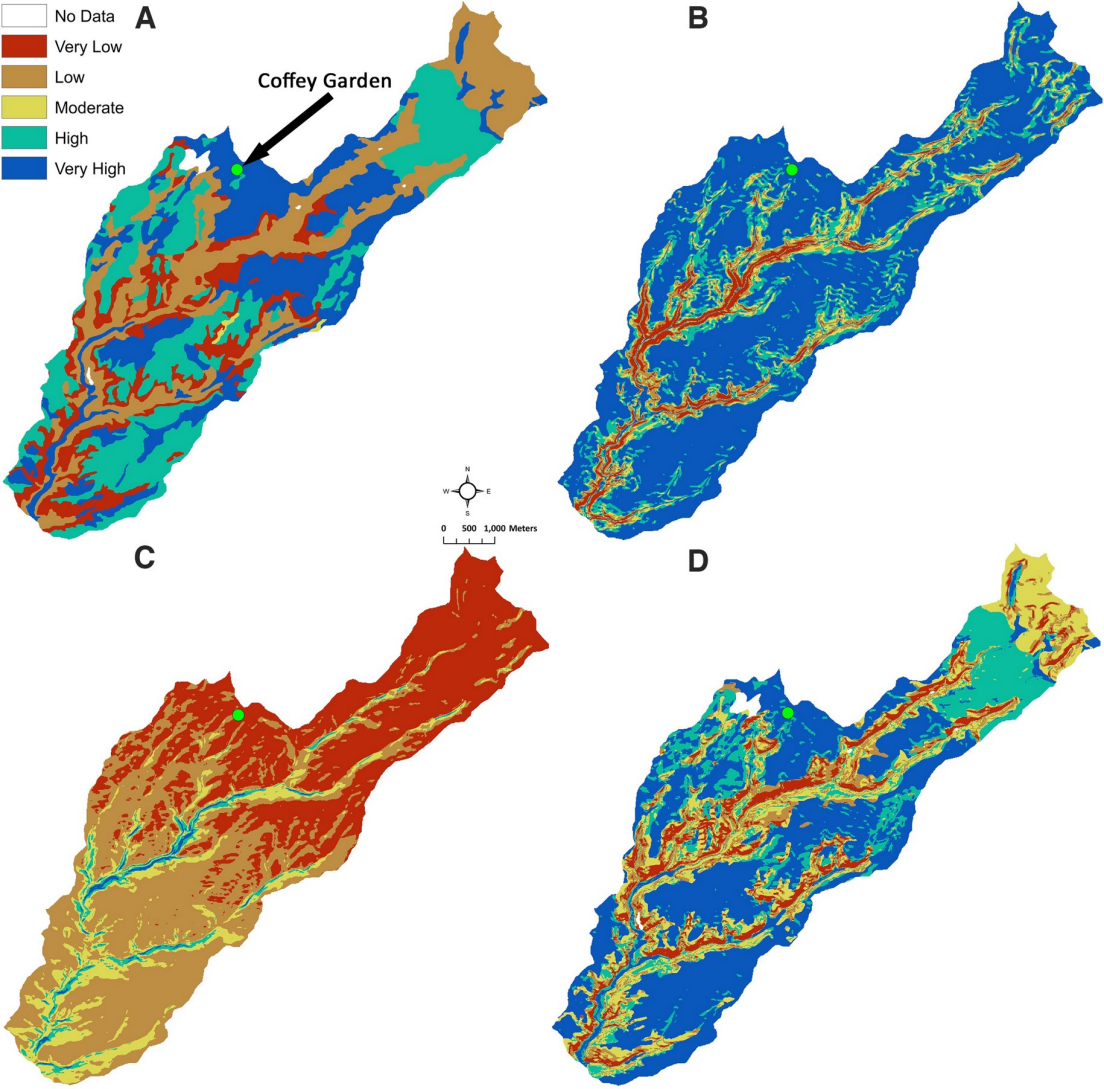
Classification of the three variables used in the SMPM and the SMPM results for the Coffey watershed. (A) Reclassification of plant available water (PAW). (B) Reclassification of slope. (C) Reclassification of solar radiation (growing season). (D) Soil moisture proxy model results. The dot towards the center and top (also shown by arrow in A) represents the location of the Coffey garden.

The final sensor was placed in a bean field called the Coffey garden (Fig 1), which is in the Coffey watershed (elevation = 1980–2300 m) in the Montezuma Subbasin, approximately 40 km N by W from Goodman watershed and at an elevation of approximately 2230 m (Fig 1). The Coffey watershed appears to be quite different from the other two watersheds (Fig 6). For the Coffey garden, the SMPM output reveals that the garden is within a very high potential soil moisture area (Fig 6D). The soil moisture data show that when all depths are averaged or whether depths are treated individually at each soil moisture location that the Coffey garden generally has the highest amount of volumetric moisture content in our study (Table 4). In addition, soil temperature is two to three degrees cooler than at the Crow Canyon gardens, meaning that evapotranspiration is potentially lower at the Coffey garden (Table 4). The temperature difference is likely due to the higher elevation of the Coffey garden. From the Coffey sample, we were able to see differences across watersheds when the settings are different. With the data from Tables 5 and 6, we can see that the Coffey watershed probably has better soil moisture potential than Crow Canyon, thus, the comparison shows that the geographic scale of the model application matters.

**Table 6:** Maximum and minimum values for SMPM variables in the Crow Canyon, Coffey, and Goodman watersheds. PAW is in cm, slope is in percent rise, and solar radiation is in WH/m^2^.

**Crow Canyon Watershed**		
	Minimum	Maximum
PAW	7.74	27.68
Slope	0.00	96.97
Solar Radiation	500,508.44	780,842.50
**Coffey Watershed**		
PAW	3.41	27.68
Slope	0.00	107.87
Solar Radiation	511,786.81	824,121.63
**Goodman Watershed**		
PAW	1.02	27.68
Slope	0.01	173.10
Solar Radiation	340,845.16	801,829.69

At an ordinal scale, the model corresponds with sensor data. The results from the three watersheds and the sensor data indicate that the analyses are strongest for evaluating intra-watershed variability. The SMPM employs the assumption that the model inputs (i.e., slope, solar radiation, and PAW) are constant across space. To compare across watersheds, a researcher would need to consider the input values for those locations, which would provide a comparative ranking at best. For example, PAW and slope values are relatively similar in the Crow Canyon and Coffey watersheds; however, solar radiation has a greater maximum in the Coffey watershed compared to Crow Canyon watershed (Table 6). Moreover, the soil temperatures are slightly lower in the Coffey watershed. In addition, it is likely that another variable is affecting the microclimate—greater precipitation at higher elevation.

It may seem appropriate to compare the experimental gardens and watersheds in a single analysis. Ultimately, however, the choice of what scale to model is dependent upon the research question and the spatial resolution of data. Scaling up the analysis to include a whole region here would not be appropriate, because water is mostly contained within (or at least exiting at single points of) multiple larger watersheds. At larger watershed or basin scales, it would be difficult to control for differences in topography and to make choices about the number of classes for each variable. For example, if a researcher is interested in large areas, such as would be required to understand soil moisture potential across McElmo and Montezuma basins, then it would be appropriate to first model for the entire area to explore what smaller subbasins might be of interest.

The Pueblo Farming Project has continued to collect information on yields and measurements on crops within the experimental gardens for several years [17]. The yield information could potentially provide another line of evidence for ground-truthing our model. The hypothesis would be that the garden with generally higher moisture content should also have higher yields, although other factors such as soil fertility and cold air drainage may prove to be other important factors when considering yield data [17,151–153].

## Discussion and conclusion

The SMPM has several improvements from previous models. First, the methodology we developed is simple and user friendly. We focus on one summary variable, soil moisture, which has predictable hydrological processes that affect it. Thus, there are fewer assumptions that need to be made. The GIS methodology is also simple. By weighting all three variables (slope, solar radiation, and PAW) equally, we can see the role each variable plays in the output. The datasets for the three variables we use are free and publically available in the United States. In addition, they are recorded at a relatively high spatial resolution (10 m), except for the limitations on the soils data, at a scale that is relevant to understanding what likely occurred across the prehistoric agricultural landscape. Because watersheds are scalable, the methodology can be scaled up to slightly larger watersheds, or scaled down to sub-watersheds or a subset of a watershed as we have done here. It should be noted that the larger the area covered, the more variability that is likely to be represented by the three variables. Thus, more than five ranked classes of soil moisture may be required. We do not recommend using our model for large regions, particularly ones that subsume multiple watersheds or high sub-watershed variability. However, one could conduct an exploratory inter-watershed analysis by comparing values across sub-watersheds, as discussed above.

The SMPM differs from other models in that it characterizes retention of water in the soil column. In effect, the model describes the variability across the watershed for the time it will take for the soil to move from field capacity to wilting point in the event of no precipitation or no input of water into the system. Thus, areas identified in the very low category are more likely to reach wilting point during times of low precipitation or drought before areas of very high soil moisture potential. Looking at the whole watershed then, the proportion of area in these different soil moisture categories can be useful for understanding the potential impacts of climate change on agriculture. Large areas of low soil moisture potential would suggest that the watershed would be more vulnerable to the effects of drought and thus crop failure. Relative to each watershed, the Goodman watershed would be the most susceptible as it has the highest percentage (29.1%) of low and very low soil moisture areas (Table 3). The Crow Canyon and Coffey watershed would be less susceptible, which have 15.3% and 20.2%, respectively, of low and very low soil moisture areas.

The watershed can also be characterized in terms of the patchiness of the distribution of higher soil moisture areas. Large contiguous areas of higher soil moisture potential would mean that fields could be in certain areas to mitigate crop loss, whereas smaller dispersed patches would suggest that fields must be smaller or there would be greater likelihood of crop loss. In the Goodman watershed, the very high soil moisture areas are associated with the south side of small creek slopes. There are a variety of ways to measure patchiness (e.g., FRAGSTATS) [154]; however, further examination of this topic is beyond the scope of this paper.

The model can also be used to understand the impacts of springs or water diversion features on the hydrology of an area. Ancestral Pueblo farmers used a mixture of techniques consisting of dryland farming and controlled surface runoff that relied on management of the landscape to redirect water to a desired location. As with patchiness, the study of soil moisture in geographic relation to springs and water-diversion features is beyond the scope of this paper, but there is high potential for future research on this topic using our model.

Outputs from our model can be subjected to varying levels of interpretation; a more comprehensive hydrological model would provide specific soil moisture values. Despite the ordinal (ranked) scale of our model, we propose that areas belonging to the high and very high potential soil moisture ranks would have had high potential for ancient farming. The collected soil moisture data from experiments in the Crow Canyon gardens reveal potential values that we could expect in a given location if the conditions are relatively similar (i.e., similar slope percentage, solar radiation, and PAW values). Validation of our model could be strengthened with direct soil moisture measurements from a broader diversity of geographic contexts.

**Fig 7:**
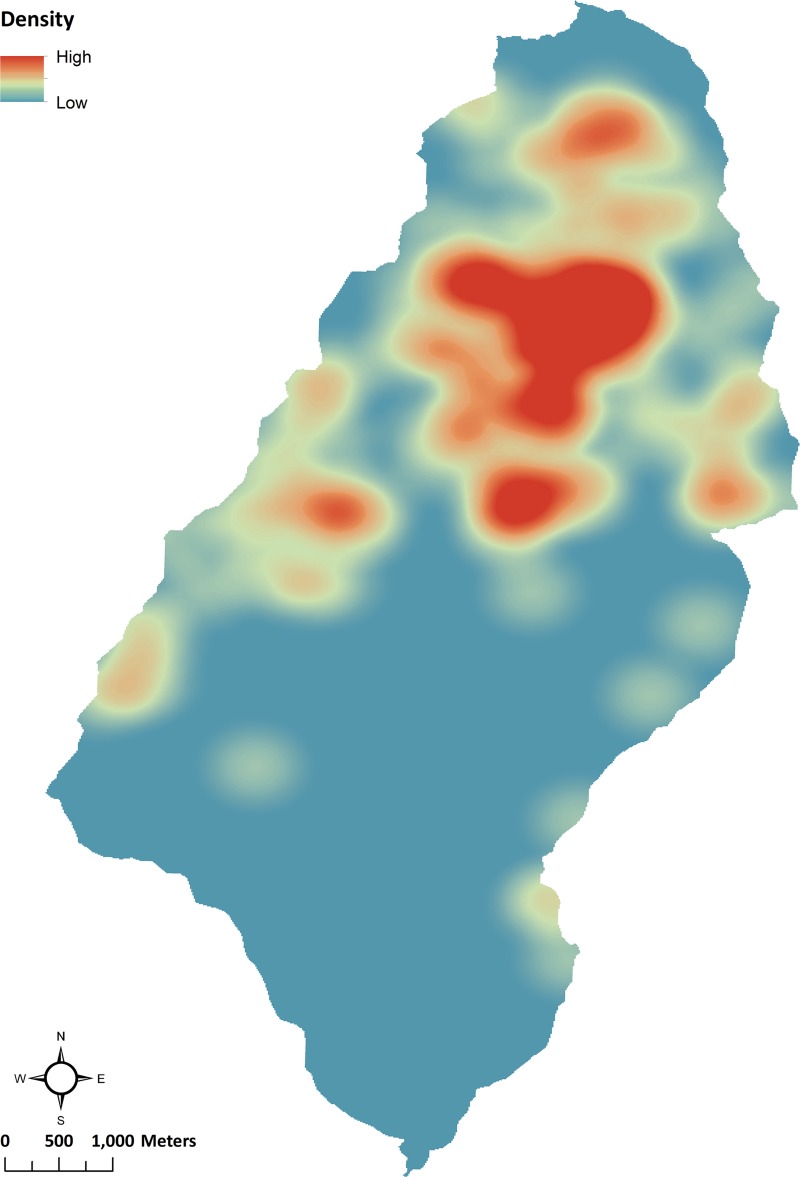
Settlement density in Goodman watershed. Kernel density of 130 archaeological settlements in the Goodman watershed dating to AD 500–1285.

If Ancient Pueblo farming was more common in areas that we found ranked high and very high for soil moisture, one expectation is that villages should be located in proximity to those areas. The density of archaeological settlements is highest in areas with higher soil moisture, particularly in the northern and western part of Goodman watershed (Fig 7) [5,7,155–157]. A Kolmogorov-Smirnov test (α = 0.001) was run to determine whether two independent samples, soil moisture class and archaeological sites (n = 130), were drawn from the same population. The maximum difference (D) between the proportion of soil moisture and proportion of archaeological sites in each soil moisture class attained is 0.2854, which is sufficient to reject the null hypothesis (Table 7). We conclude that the relationship between soil moisture classes and site location in this sample of archaeological sites is significant.

**Table 7:** Kolmogorov-Smirnov test for soil moisture class and archaeological site location.

Soil Moisture Class	Proportion of Total Area	F_o_(X)	S_n_(X)	D_n_
1	0.125	0.125	0.0308	0.0942
2	0.166	0.291	0.0462	0.2448
3	0.256	0.547	0.2616	0.2854
4	0.309	0.856	0.7308	0.1252
5	0.144	1	1	0

New archaeological evidence brings additional insight to how our model might be useful for the study of ancient farming [158]. Crow Canyon Archaeological Center excavated portions of the Goodman Point Unit, located in the northern portion of the Goodman watershed, from 2005 to 2011, and they also conducted a soils and geomorphological survey at that time. In one of the patches that had a high and very high soil moisture rank (4–5), maize pollen was discovered in two test units. These units are in a meadow that has no archaeological sites. Additionally, test units that fell into the moderate soil moisture class did not yield maize pollen. Maize pollen can travel up to approximately 800 meters, but the pollen typically stays within 60 meters of the plants [159,160]. We suggest that additional soil tests for maize pollen could be conducted to determine whether maize primarily occurred within those areas with high and very high soil moisture potential as identified by our model.

The utility of the model will depend on the variability in the landscape because the classification that the model generates is relative to the sample area. If too large of an area is studied and if topographic and soils variability is high across space, then the high and very high soil moisture areas may be smaller or patchier in distribution. For example, if we were to enlarge the study area to the McElmo watershed, then the very high soil moisture areas might be in different locations in the Goodman watershed. That is, increasing the area, changes the sensitivity of the model and thus the resolution of soil-moisture-potential mapping. It is also possible that no very high soil moisture patches would be identified in the Goodman watershed. In contrast, if the study area is too small or if the area is relatively homogeneous, then most of the landscape would have a similar rank or very small differences might become exaggerated.

It is important to reiterate that the appropriate spatial scale of the model depends on the research question. If the goal is to describe or characterize a larger watershed, then a lower resolution model might be appropriate. Our assumption is that even for larger areas, we should see archaeological sites near moderate to very high soil moisture areas. Within a watershed, a high proportion of sites should be near high to very high soil moisture patches. If the goal is to understand agricultural potential, then the sub-watershed scale might be more appropriate because people are less likely to select fields far away from habitation sites (e.g., one or more sub-watersheds away).

The model allows for simplification of the complex process of soil moisture by isolating key variables. By spatially modeling the soil moisture proxy, the landscape can be viewed as a mosaic of soil moisture patches. This can be applied to an archaeological setting by considering the high to very high soil patches to be high potential farmland in the watershed. Application of the model provides a better understanding of how landscape dynamics within the watershed affected Ancestral Pueblo communities. The Ancestral Pueblo people were likely selecting site locations based on the distance to the best farmable lands, which in this context was likely to have been connected to the limiting variable of soil moisture.
